# A Droplet Microfluidics Based Platform for Mining Metagenomic Libraries for Natural Compounds

**DOI:** 10.3390/mi8080230

**Published:** 2017-07-25

**Authors:** Elias Theodorou, Randall Scanga, Mariusz Twardowski, Michael P. Snyder, Eric Brouzes

**Affiliations:** 1 Metagenomix Inc., Branford, CT 06405, USA; mariusz.t@gmail.com; 2 Department of Pathology, Yale University School of Medicine, New Haven, CT 06510, USA; 3 Department of Chemistry, Massachusetts Institute of Technology, Cambridge, MA 02139, USA; randallscanga@yahoo.com (R.S.); mariusz.t@gmail.com (M.T.); 4 Department of Genetics, Stanford University School of Medicine, Stanford, CA 94305, USA; 5 Department of Biomedical Engineering, Stony Brook University, Stony Brook, NY 11794, USA; 6 Laufer Center for Physical and Quantitative Biology, Stony Brook University, Stony Brook, NY 11794, USA

**Keywords:** metagenomic screening, droplet microfluidics, high-throughput screening, natural compound

## Abstract

Historically, microbes from the environment have been a reliable source for novel bio-active compounds. Cloning and expression of metagenomic DNA in heterologous strains of bacteria has broadened the range of potential compounds accessible. However, such metagenomic libraries have been under-exploited for applications in mammalian cells because of a lack of integrated methods. We present an innovative platform to systematically mine natural resources for pro-apoptotic compounds that relies on the combination of bacterial delivery and droplet microfluidics. Using the violacein operon from *C. violaceum* as a model, we demonstrate that *E. coli* modified to be invasive can serve as an efficient delivery vehicle of natural compounds. This approach permits the seamless screening of metagenomic libraries with mammalian cell assays and alleviates the need for laborious extraction of natural compounds. In addition, we leverage the unique properties of droplet microfluidics to amplify bacterial clones and perform clonal screening at high-throughput in place of one-compound-per-well assays in multi-well format. We also use droplet microfluidics to establish a cell aggregate strategy that overcomes the issue of background apoptosis. Altogether, this work forms the foundation of a versatile platform to efficiently mine the metagenome for compounds with therapeutic potential.

## 1. Introduction

Over the last two decades there has been a gradual decrease in the number of new chemical entities, that is, drugs with completely novel chemistries. Though significant advances have been made with combinatorial chemical synthesis, these yield compounds inferior to those found in nature which have evolved to bind protein motifs that are conserved throughout the animal kingdom. A potentially rich source of natural compounds is found within complex communities of microbial organisms but shortcomings of current technologies severely limit the efficient screening of those biomasses.

The identification of natural compounds produced by microbes isolated from various environments has led to the discovery of medically relevant compounds such as rapamycin and FK-506 (tacrolimus) [1,2,3]. However, more than 99% of microbes cannot be cultured, which is a pre-requisite to obtain screen-able quantities of bioproducts [4,5,6]. To gain access to this so-called “dark matter”, a variety of approaches have been undertaken including the development of specific growth media [7,8], the isolated growth of microbes within a gel emulsion [9] or a diffusion chamber [10]. Overall, these strategies introduce a significant bias by selecting for a narrow range of species able to grow in such conditions.

Circumventing culture of microbes by extracting metagenomic DNA has become the most prevalent method for mining useful enzymatic activities and compounds from large communities of uncharacterized microbes [11,12,13]. Purified metagenomic DNA is used to generate plasmid libraries, which are then propagated and expressed in bacterial strains with simple growth requirements. Bio-active compounds or enzymes are then produced and isolated before testing in different assays. This approach has permitted the isolation of compounds with potential clinical value [14,15] but the majority of screens have focused on identifying enzymes with industrial value [16,17,18]. A major limitation in the current methodologies used for screening metagenomic libraries in mammalian cells is that extracts must be prepared and tested in a multi-well format. Added to the challenge is the inability of any single extraction agent to act as a universal solvent for all novel compounds [19], necessitating repeated extractions and screenings for each library. Such an approach is laborious and greatly limits the number of clones and compounds that can be screened. Critically, there have been limited efforts to screen metagenomic libraries in mammalian cells because the number of bio-active compounds in functional screens can be extremely low [15,20]. Translating those screens to mammalian cells would entail the preparation and testing of hundreds of thousands or millions of clonal extracts in a multi-well format to identify a handful of lead compounds. Recently, bio-informatics approaches relying on deep sequencing and selection for the signature of clustered enzymes have yielded some success outside of functional screening, but these methods generally depend on known or predicted peptide motifs [21,22].

To overcome these issues, we have developed a bacterial-based method for identification of novel metabolites that bypasses the large-scale automation as well as the extract preparation needed for delivering metagenomic-based drugs directly into mammalian cells. Combined with droplet microfluidic technology, our platform (1) reduces the costs associated with standard screening practices, (2) eliminates the need for multi-well plates, and (3) can be utilized to identify natural products that produce desired in vitro effects on an unprecedented scale.

## 2. Materials and Methods

### 2.1. Plasmid Construction and Generation of E. Coli Strains

Plasmid TRIP 3.0 (Figure S1) was designed with the invasin ORF driven by a constitutive promoter. The Listeriolysin O (LLO) gene and an additional ribosome binding site (RBS) were incorporated immediately downstream of invasin that confers invasive properties to bacteria (see main text). The presence of the RBS allowed for the elimination of a large amount of upstream and downstream sequences that were retained in a previously published plasmid dependent on multiple promoters [23]. A mid-copy origin of replication, pACYC, was incorporated to avoid recombination between TRIP 3.0 and the origins of any additional plasmids co-transformed into our *E. coli* strain of choice DH10B.

The violacein operon (VioA-E) was cloned into the pUC19 vector (New England Biolabs, Ipswich, MA, USA) and used to transform DH10B (ThermoFisher Scientific, Waltham, MA, USA) cells previously transformed with plasmid TRIP 3.0 and maintained in growth media containing 50 μg/mL kanamycin and 100 μg/mL carbenicillin for dual expression of invasin and violacein. Chemically competent DH10B cells were prepared by calcium chloride treatment [24].

DH10B double positive for pUC19-VioA-E and TRIP 3.0 were transformed with spectinomycin resistant inducible vector pJExpress T5-lacO RFP for labeling cells with red fluorescent protein. RFP was produced constitutively by maintaining 1 mM Isopropyl β-d-1-thiogalactopyranoside (IPTG, Sigma-Aldrich, St. Louis, MO, USA) in all media from the time of bacterial colony inoculation through mammalian cell invasion and cell culture.

### 2.2. Cell Culture

HeLa S3, African green monkey kidney (Vero) cells and P19 embryonic carcinoma (P19 EC) cells were obtained from American Type Culture Collection (ATCC). Cells were incubated at 37 °C in humidified 5% CO_2_ atmosphere in DMEM supplemented with 25 mM HEPES 2 mM l-glutamine and 10% Fetal Bovine Serum (all reagents from ThermoFisher Scientific) in antibiotic-free conditions. 

For mammalian cell infection, we followed guidelines from Hicks et al. [25]. Briefly, HeLa S3 or Vero, cells were plated at 6–8 × 10^5^ cells/well in 6-well plates 24 h prior to infection. Bacteria were diluted to the desired Multiplicity of Infection (MOI) in cell culture media and overlaid on target cells. Plates were spun at 1000× *g* for 30 min at room temperature and then incubated for 1 h at 37 °C. Following infection, mammalian cells were washed several times in PBS containing 1000 μg/mL gentamicin (Sigma-Aldrich), incubated in media containing 100 μg/mL gentamicin, and finally switched to media containing 10 μg/mL gentamicin for the remainder of the assay.

For intracellular bacteria counts via gentamicin protection assay, mammalian cells were washed three times with PBS + 100 μg/mL gentamicin, lysed with 0.5% Triton X-100 for 10 min, and dilution series were plated onto LB agar. Mammalian cells but not the bacteria were lysed by this treatment [26,27]. Visualization of droplet encapsulated cells, cell staining, and bacterial-mammalian interactions were performed on a Nikon Ti-S Inverted Microscope L100 for phase contrast and fluorescence images. Image capture was performed with a QICAM Fast 1394, mono, 12-bit cooled digital camera (QImaging, Surrey, BC, Canada) using Nikon Elements software (Nikon, Tokyo, Japan).

### 2.3. Apoptosis Experiments

Live cells were stained with annexin V-fluorescein isothiocyanate (Annexin V-FITC Apoptosis Kit, CalBiochem). Stock solutions were diluted directly into cell culture media and cells returned to humidified CO_2_ incubators for a period of one hour at 37 °C. FAM Vybrant caspase reporter peptides (ThermoFisher Scientific) were used according to manufacturer’s specifications.

### 2.4. Purified Violacein Experiments

Purified violacein from *Janthinobacterium lividum* (V9389, Sigma-Aldrich) was solubilized in absolute ethanol for cell culture assays.

### 2.5. Microfluidic Chip Fabrication

Microfluidic chips were hybrid PDMS/glass chips fabricated using soft-lithography [28,29] by the University of Utah microfluidics foundry. The designs (File S1) were prepared with AutoCAD software and printed at 25,400 dpi resolution onto a Fuji transparency mask (CAD/Art Services Inc., Bandon, OR, USA). Upon reception, the microfluidic chips were treated with a fluorinated trichloro silane reagent (heptadecafluoro-1,1,2,2-tetrahydrodecyl) trichlorosilane-CF_3_-(CF_2_)_6_-CH_2_-CH_2_-SiCl_3_, Gelest, PA, USA) diluted at 1% weight in HFE7500 oil (3M) [28]. The solution was injected into channels with a disposable syringe, through a hydrophobic 0.2 μm disc filter and a blunt needle, and flushed out with FC 3283 (3M, Maplewood, MN, USA) oil after 5 min of incubation. We injected liquid low-melting solder (Cerrolow-117, 47 °C melting temperature, Bolton Metal Products, Bellefonte, PA, USA) in dedicated channels while the chip was incubated on a hot plate to fabricate the electrodes. A piece of copper foil tape with conductive adhesive was affixed to the back of the chip to confine the effects of the electric field.

### 2.6. Surfactant and Oil System

We used a PEG-derived krytox (Dupont, Wilmington, DE, USA) surfactant dissolved at 1% weight into HFE7500 fluorinated oil (3M). We synthesized the surfactant as described previously [30]. We added a destabilizing co-surfactant 1*H*,1*H*,2*H*,2*H*-Perfluoro-1-octanol (370533, Fluke, Everett, WA, USA) to disrupt emulsions in bulk after removing most of the fluorinated oil. Upon emulsion disruption, the aqueous content was in the upper phase and easily recoverable.

### 2.7. Microfluidic Set-Up

The microfluidic set-up was based on an inverted microscope (IN200AB, Amscope) equipped with a Firewire camera (XCD-V60, Sony, Tokyo, Japan). The illumination was accomplished with a high-power LED (Luxeon) whose intensity was manually controlled with a buckpuck. Fluids were actuated with a pressure-driven system (MFCS-4C-7000mbar, Fluigent, Paris, France), after being loaded into 15 mL tubes equipped with caps (Fluiwell, Fluigent) that serve as port to connect 1/32” peek tubing (Idex) whose other extremity was directly inserted in chip inlets or outlets. Cells were maintained as a homogeneous suspension using a Stirring Bottle (neMIX, Cetoni, Germany). The high-voltage high-frequency electric field necessary for the merging module was delivered by a Cold Cathode Fluorescent Lamp inverter (BXA-12579, JKL Components Corp.) regulated by a DC power supply (1686A, B&K Precision). The high-speed movies were obtained using a high-speed EX-F1 camera from Casio capable of 1200 images per second, which was mounted onto an eyepiece using an adapter (http://www.cncsupplyinc.com).

### 2.8. Preparation of Droplet Samples for FACS Analysis

Following incubation after library merging, the droplet emulsion was disrupted in bulk by adding a destabilizing co-surfactant (perfluoro-octanol) to the oil phase to allow harvesting of HeLa S3 cells that were stained for apoptosis and analyzed by cytometry. Recovered cells were washed multiple times and incubated in gentamicin containing cell media during the apoptosis incubation phase. During the last hour of a 4-h incubation, FLICA-555 was added to the cell culture media. Post-incubation cells were washed to remove excess FLICA peptide, frozen and stored at −80 °C until thawed for fluorescent activated cell sorting. Flow cytometry was performed with a FACS Aria instrument (Becton Dickinson, Franklin Lakes, NJ, USA) at the core facility of Northwestern University (Evanston, IL, USA).

### 2.9. Preparation of Samples for COPAS Sorting

P19 EC aggregates recovered from disrupted droplets after bacterial infection were washed by allowing their sedimentation at room temperature, followed by several washes with PBS containing 80 μg/mL gentamicin, further incubated in DMEM culture media with 80 μg/mL gentamicin for one hour and finally resuspended in DMEM media. To avoid clumping of aggregates during overnight incubation with intracellular bacteria, ultraPure agarose (ThermoFisher Scientific) was solubilized in DMEM media by heating in a microwave to 0.1% (*w/v*), followed by the addition of FBS and 10 μg/mL gentamicin. 100 μg/mL carbenicillin was added to prevent potential loss of violacein plasmid from intracellular bacteria. After overnight incubation at 37 °C, P19 EC aggregates were harvested by a 2-fold dilution of agarose containing incubation buffer with DMEM media followed by centrifugation at 100× *g* for 5 min, collecting the bottom 3 mL of solution and aggregates, further diluting in DMEM media and allowing aggregates to sediment before bringing up in DMEM media. P19 EC aggregates were kept in DMEM media on ice for up to several hours prior to sorting. They were incubated with 10 mM propidium iodide (ThermoFisher Scientific) for 10 min at room temperature, during which aggregates were allowed to sediment, before resuspension in ice cold PBS Ca^2+^ Mg^2+^ and sorting at 50 events per second into media with a COPAS Biosort [31] (Union Biometrica, Holliston, MA, USA). 2X Triton lysis buffer was added to sorted aggregates before plating onto agar supplemented with 100 μg/mL carbenicillin. 

## 3. Results

### 3.1. Strategy

We designed a platform where natural compounds produced from biosynthetic clusters can be directly delivered by infectious bacteria into mammalian cells to bypass laborious extraction steps. The bacteria combine three critical properties by serving as cloning, production and delivery agents. Interestingly, not all invasive bacteria lyse within mammalian cells and some intact bacteria can be isolated, and their plasmid DNA extracted even after overnight incubation. These intact bacteria provide a straightforward way to determine the coding sequence of the metabolic pathway of active bio-compounds as well as a renewable source of product. The screening format is based on droplet microfluidics where aqueous samples are encapsulated into droplets that serve as independent reactors [32,33,34,35,36,37,38,39,40]. The droplets are generated in oil, stabilized by a surfactant, and can be manipulated at a rate of thousands per second. In our workflow, droplets fulfill several important roles: (1) their ability to be handled at rates of thousands per second allows for a high-throughput screening platform; (2) droplets act as isolated vessels for the amplification of metagenomic libraries with minimal bias; (3) droplets allow for clonal dosing of mammalian cells with drug producing bacteria, where a mammalian cell is infected with several individual *E. coli* each producing the same drug; and (4) droplets allow for the controlled generation of cell aggregates which serve as in vitro targets, an approach that minimizes the impact of the rate of false-positives, which is critical when screening a large library for rare toxic compounds.

**Figure 1:**
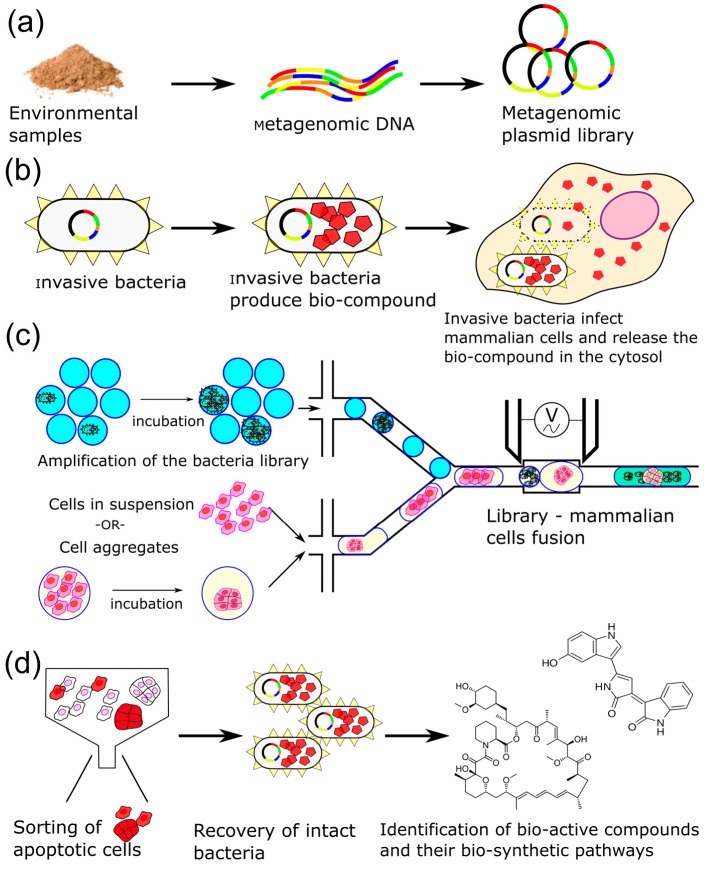
Overall strategy to mine natural resources for pro-apoptotic bioproducts. (**a**) Generation of metagenomic libraries from environmental samples; (**b**) Delivery of bioproducts by invasive bacteria into mammalian cells. Invasive *E. coli* producing a natural compound are taken up by mammalian cells and are lysed in phagosomes. The released natural product (red pentagon) is then free to interact with its targets; (**c**) Droplet microfluidics is critical by enabling: (1) the unbiased amplification of the metagenomic library by limited encapsulation of a single bacterium, (2) the controlled generation of cell aggregates, and (3) the maintaining of clonality of the entire process; (**d**) After treatment, apoptotic cells or aggregates are sorted, pro-apoptotic invasive bacteria are recovered and their plasmid isolated. In parallel, bacteria can be analyzed by mass spectrometry to identify the bio-active compounds.

In detail, metagenomic DNA is first extracted from environmental samples and inserted into high, mid, or low copy plasmids to create a metagenomic library (Figure 1a). The metagenomic DNA is size selected to ensure a fragment length with enough protein coding regions (colored bars) to encompass an entire biosynthetic pathway capable of producing a natural product. Metagenomic DNA is then inserted into a plasmid expression vector and subsequently transformed into a nonpathogenic laboratory strain of bacteria such as DH10B *E. coli* that has been rendered invasive (Figure 1b).

The bacteria library is amplified after single-bacterium encapsulation which allows for the maintenance of library complexity by avoiding fitness competition between clones. The library amplification step is also necessary to maintain clonality of the bacteria in the workflow, allowing micro-batches of mammalian cells to be dosed with a single type of drug producing bacteria. This step hinges on the stability of the droplets and low toxicity of the fluorinated oil and surfactant [30]. Droplets containing amplified metagenomic clones are then combined with droplets containing either mammalian cells free in suspension or pre-aggregated via incubation in permissive conditions. After electro-fusion of the mammalian and bacterial droplets, a short incubation allows bacteria to invade mammalian cells (Figure 1c). Some of the bacteria will be lysed within the endocytic vesicles, releasing their contents, including any newly synthesized bio-active compounds, into the cytosol. Other bacteria remain viable, allowing for their recovery and for the identification of the biochemical pathway responsible for the synthesis of the bio-active compound. Mammalian cells that have been exposed to pro-apoptotic metabolites can be identified by the cleavage of fluorescent cell-permeable reporter peptides specific for caspase 8 or 9, proteases active in early stage apoptosis. After droplet disruption and separation of mammalian cells from any remaining extracellular bacteria, apoptotic cells are isolated by fluorescence activated cell sorting (FACS). Cells are immediately lysed with a mild detergent and the viable bacteria are recovered to determine the structure of the natural product by mass spectrometry and their metagenomic DNA fragments are sequenced to identify the associated biosynthetic pathway (Figure 1d). The outlined approach is an efficient strategy to mine the vast metagenome for natural compounds that are active in mammalian cells.

### 3.2. Extraction-Free Method for Mammalian Cell Delivery of Microbially Produced Natural Products

**Figure 2:**
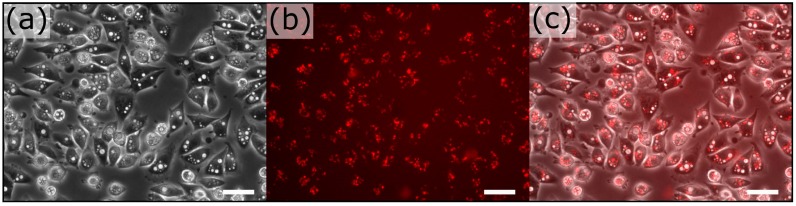
Bacterial delivery of violacein at MOI of 25 at 4 h post-infection. (**a**) Phase contrast image exhibits the formation of large vacuoles within most cells; (**b**) Red channel exhibits invasive bacteria that harbor a RFP reporter; (**c**) Overlay indicates an abundant number of internalized bacteria for downstream retrieval. Scale bar represents 50 μm.

Our metagenomic drug screening technology relies on an efficient means to deliver bacterially produced natural compounds into mammalian cells regardless of the extraction and solubility requirements of such compounds; a major hurdle for high-throughput screening of metagenomic libraries in mammalian cells. The method involves a simple modification of a bacterial strain to assure the release of the novel product directly within the target mammalian cell (Figure 1b). To accomplish this, we generated the plasmid TRIP 3.0 which incorporates the coding sequences for invasin from *Y. pseudotuberculosis* to impart invasive capability and listeriolysin O from *L. monocytogenes* to assure efficient delivery [41,42] (Figure S1). As previously reported [23,43], the *Y. pseudotuberculosis* protein invasin confers on *E. coli* the ability to invade both dividing and quiescent mammalian cells. Using a gentamicin protection assay [44] we found that *E. coli* strain DH10B constitutively expressing invasin could efficiently invade two different mammalian cell lines—Vero African green monkey kidney epithelial cells and HeLa S3 human cervical carcinoma cells (Figure 2). Observation of HeLa S3 dosed with invasive and red fluorescent DH10B indicated the integrity of numerous internalized bacteria that could be recovered for further analysis (Figure 2).

We then tested the ability of invasive bacteria to deliver natural products with a fragment of the *Chromobacterium violaceum* genome encompassing the violacein operon. Violacein is a pigmented antibiotic which gives *C. violaceum* colonies a dark violet color. The production of violacein evolved as a defense for *C. violaceum* biofilms and phagocytosis of a single *C. violaceum* results in lysis, release of violacein, and induction of apoptosis in protozoa [45]. Additionally, violacein has been shown to be toxic not only to protozoa but also to induce apoptosis in a range of human cancer cell lines [46,47,48,49,50]. Furthermore, violacein is insoluble in water [51] and thus fits our model of identifying natural compounds that may or may not require specific solvents for mammalian cell assays. Violacein production in *E. coli* is also straightforward because the violacein operon is sufficient for the synthesis of violacein in the presence of tryptophan and oxygen substrates [52]. Therefore, we cloned the violacein operon into the high copy pUC19 vector with a minimal constitutive kanamycin resistance promoter and co-transformed it with plasmid TRIP 3.0 into DH10B *E. coli*. Violacein production was evidenced by the dark violet colonies of dual transformants grown on selective media (Figure S2).

**Figure 3:**
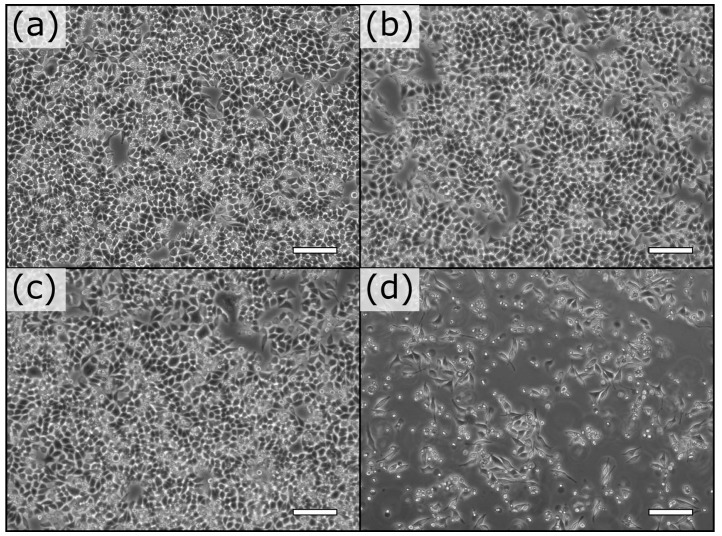
Effect of bacterial delivery of violacein on HeLa S3 cells at MOI of 10. At 4 h post-treatment, there is no observable effect on HeLa S3 cells treated with bacteria expressing empty vector (**a**); vioA-E operon (**b**); invasin (**c**); but internalization of vioA-E (**d**) resulted in the loss of 65% of the cells. Scale bars represent 100 μm.

Dual transformants DH10B were tested for their ability to invade and induce apoptosis of mammalian cells. Overnight bacterial cultures were diluted and spun onto monolayers of near confluent target cells. We observed that invasive bacteria expressing violacein resulted in detachment of most (70% compared to controls) HeLa S3 cells within 20 h even at a low multiplicity of infection (MOI) of 10 (Figure 3d versus Figure 3a–c). Comparable results were obtained for Vero cells with up to 90% of treated cells detaching compared to controls (Figure S2). Control bacteria expressing invasin demonstrated mild toxicity in the form of detachment at a MOI of 75, well above the minimal effective MOI needed for widespread infection.

Observation of HeLa S3 dosed with violacein producing DH10B cells indicated the widespread presence of vacuoles at 4 h post-infection for MOIs of 10 and 25 (Figure 2). Because the vacuoles were specifically induced by violacein [53], their presence was used as an indicator of its effective delivery in our system.

**Figure 4:**
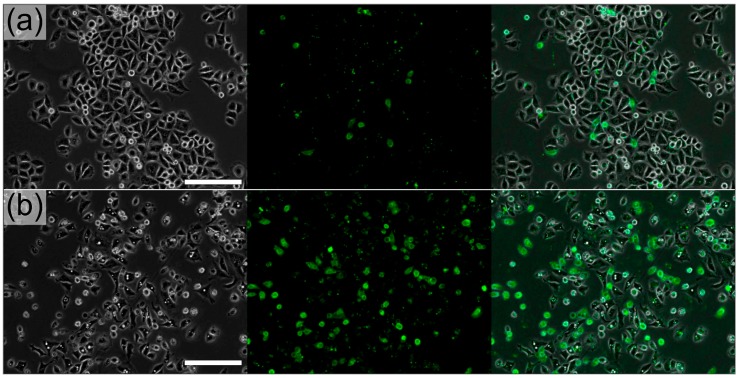
Annexin V staining demonstrates the apoptotic effect of bacterially delivered violacein at a MOI of 25 at 10 h post-infection. (**a**) 7% of HeLa S3 cells treated with a control invasive DH10B showed annexin V staining (right panel); (**b**) 33% of HeLa S3 cells treated with bacterially delivered violacein showed annexin V staining. Scale bars represent 100 μm.

Live cells were labeled with annexin V as an early indicator for apoptosis 6 and 10 h post-infection. A low percentage of cells stained positively for annexin V at MOI 10 and a greater percentage (33%) stained positively at MOI 25 at 10 h post-incubation (Figure 4b). HeLa S3 cells treated with a control invasive or a violacein non-invasive DH10B did not exhibit strong annexin V staining for apoptosis (7%, Figure 4a). 

This relatively rapid initiation of apoptosis after bacterial delivery of violacein was further confirmed using cell permeable Fluorescent-Labeled Inhibitor of CAspases (FAM-FLICA) peptides that specifically detect the activity of caspase 8, a marker of early stage apoptosis. HeLa S3 cells plated 24 h prior to treatment were infected at a MOI of 25 with either control invasive DH10B transformed with an empty pUC19 plasmid control or invasive DH10B transformed with pUC19-VioA-E plasmid. Following infection and removal of extracellular bacteria HeLa S3 cells were incubated for 5 h before the addition of an excess of FLICA substrate and an approximately hour-long incubation before harvesting, freezing, and subsequent thawing prior to flow cytometry. Though there was a substantial amount of background at a MOI of 25 with a total HeLa S3 treatment time of 6 h, the HeLa S3 dosed with violacein producing bacteria resulted in activation of caspase 8 to a significantly higher degree (Figure S4). Altering conditions by shortening the treatment period to 4 h, the signal to noise ratio was increased significantly, with 9.64% caspase 8 positive for control treated HeLa S3 cells and 35.48% caspase 8 positive for violacein-delivered HeLa S3 cells.

Interestingly, it was possible to recapitulate the phenotypic effect observed in HeLa S3 infected with invasin+ violacein+ DH10B using purified violacein at a concentration range of 5–10 μM after a 10 h incubation (Figure S5). The notable differences included an absence of larger vacuoles and a slower kinetics but a more uniform annexin V staining across the population (not shown). This could possibly be due to variability in the amount of violacein delivered into individual HeLa S3 cells with invasive DH10B as a vehicle.

Lastly, in establishing a screening system based on droplet manipulation, we needed to recover bacteria from mammalian cells exposed to turbulent conditions and undergoing apoptosis. Initial testing of bacterial recovery was confined to HeLa S3 cells grown on tissue culture plastic. It was determined that a comparable number of bacteria could be recovered from HeLa S3 cells at 10 h post-infection whether the HeLa S3 were infected with control invasive DH10B or infected with violacein producing DH10B. While recovery of internalized bacteria was found to be consistent for earlier time points, a concern was whether the presence of the violacein expression plasmid (or the production of any novel metabolite) would interfere with bacterial recovery because of toxicity towards the host mammalian cells. We compared the number of bacteria recovered from cells treated with invasive and non-invasive violacein producing bacteria. At 24 h post-infection with an initial MOI of 10, the number of bacteria recovered from HeLa S3 cells infected with invasive bacteria producing violacein was six-fold higher (~600 colonies) than cells treated with non-invasive bacteria. It is noteworthy that the density of adherent cells treated with invasive bacteria producing violacein was lower than control due to a rapid loss of HeLa S3 attachment to the culture vessel (Figure 3). We did not investigate this counterintuitive increase in the number of DH10B in the HeLa S3 cells undergoing apoptosis, which would have had compromised membranes. However, we speculate that the induction of apoptosis fortuitously resulted in the protection of internalized DH10B producing violacein due to the basic interruption of normal cell processes such as endosomal breakdown of the DH10B, leading to survival of intracellular bacteria. Finally, we tested the feasibility of recovering violacein producing invasive bacteria from caspase 8 sorted HeLa S3 by lysing sorted cells and plating the lysate onto antibiotic containing agar plates. Bacteria producing violacein were clearly recovered based on the dark violet pigmentation of colonies (Figure S2) and confirmed by the isolation of plasmid DNA containing the fragment of DNA coding for the violacein operon.

### 3.3. Droplet Microfluidic Workflow

**Figure 5:**
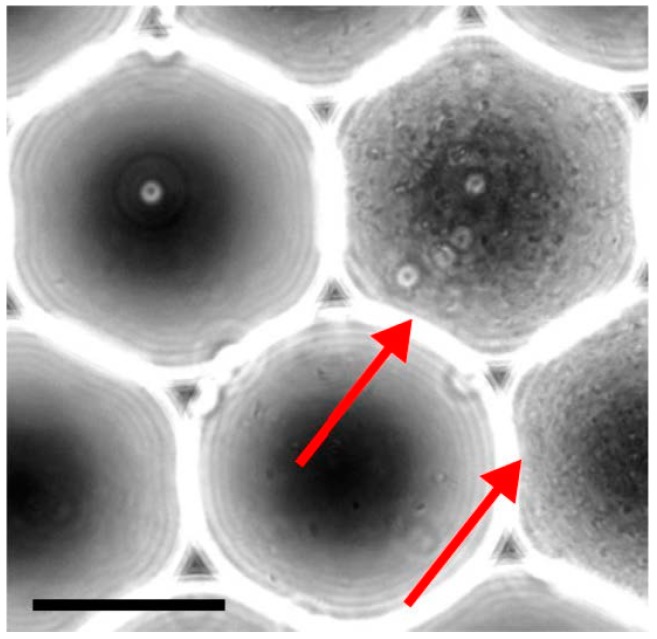
Generation of monoclonal droplets. A diluted solution of bacteria is encapsulated into 75 μm (220 pL) droplets, collected and incubated. In these specific conditions, a single bacterium is present in populated droplets while the rest of the droplets remains empty. This approach alleviates fitness competition between bacteria and maintains complexity of the initial library. Bacteria grew overnight and saturated the droplet volume (red arrows). Scale bar represents 50 μm.

Droplet microfluidics enables to amplify the library of metagenomic clones without fitness bias by encapsulating each clone in a separate reactor. In these conditions, it is expected that clone growth is only limited by the droplet volume because slow-growing bacteria do not compete with faster growing bacteria. Particle encapsulation follows Poisson’s statistics [54] and we encapsulated the library such that less than 10% of the droplets were occupied to maintain clonality. Bacteria were encapsulated into 75 μm (220 pL) droplets with LB medium and stabilized in HFE7500 oil with 1% weight PEG-krytox based surfactant using a pressure driven system and a simple nozzle design [55,56] (File S1 and Video S1). Upon overnight incubation, bacterial cell growth was readily apparent (Figure 5).

**Figure 6:**
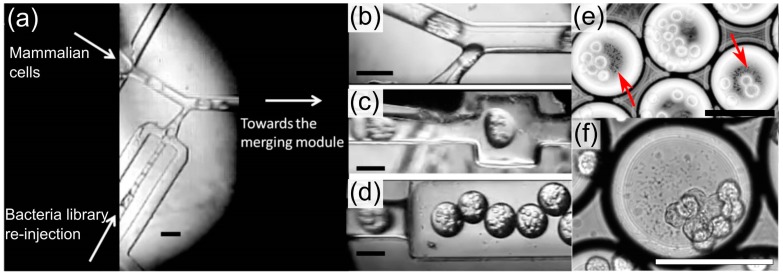
Droplet microfluidics for treatment of mammalian cells with metagenomic bacterial library. The device consists into a series of modules: (**a**) A nozzle to encapsulate mammalian cells and a nozzle to re-inject monoclonal droplets; (**b**) A fork to combine the droplet streams; (**c**) A merging module to perform pairwise electro-fusion of mammalian cells and monoclonal droplets and (**d**) a collection channel. (**e**,**f**) HeLa S3 cells co-encapsulated with bacteria (red arrows in e) at different magnification. Scale bars represent 100 μm.

We initially sought to determine whether a positive apoptosis inducing clone could be identified in the presence of background apoptosis. We dosed a suspension of HeLa S3 cells with violacein producing invasive DH10B in droplets to confirm if a drug producing operon could be detected and at least partially enriched above background in one cycle of cell sorting. We mixed invasin+pUC19-VioA-E+ DH10B and control invasin+ DH10B at a ratio of 1:1000 prior to their monoclonal encapsulation. Droplets were collected into Eppendorf tubes and incubated in a humidified 37 °C chamber overnight to allow for droplet encapsulated microcolonies to form (Figure 5). After overnight incubation, the bacteria library was re-injected and merged with freshly encapsulated HeLa S3 cells into 110 μm (700 pL) droplets using a microfluidic device comprising a merging fork followed by a fusion module [32,57] (Figure 6, Video S2). Infection of HeLa S3 cells was allowed to continue for one hour at 37 °C. Interestingly, in assays with a fluorescent protein labeled control, we observed that droplet grown invasive bacteria adhered to the surface of HeLa S3 cells within minutes (Video S2).

Following incubation, the droplet emulsion was disrupted in bulk by adding a destabilizing co-surfactant (perfluoro-octanol) to the oil phase to allow harvesting of HeLa S3 cells that were stained for apoptosis and analyzed by cytometry. Most gated events were negative for caspase 8 activity (Figure S6) but >5% were found to be positive. Approximately 10,000 caspase 8 positive and negative HeLa S3 cells were collected into sheath fluid to which 2X Triton lysis buffer was added before plating on agar plates containing antibiotics. Of 300 colonies recovered in each condition, 9 violacein positive clones were identified only in cells positive for caspase 8 activity. This experiment demonstrates our ability to isolate pro-apoptotic bacteria from background apoptosis, which is due to the infection by the invasive bacteria.

### 3.4. Cell Aggregates for Efficient Screening of Pro-Apoptotic Compounds

**Figure 7:**
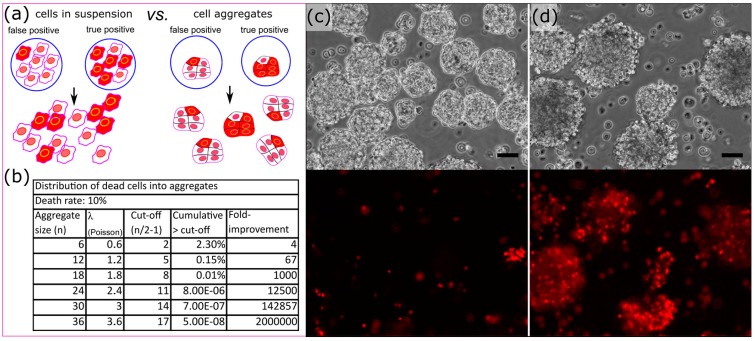
Aggregate strategy to reduce the effect of background apoptosis on the identification of bio-active compounds. (**a**) Screening the effect of rare bio-active compounds on cell aggregates reduces the noise due to background apoptosis (red: apoptotic cells); (**b**) Effect of the aggregate size on the enrichment of true positives assuming a background apoptosis rate of 10% and a sorting threshold at the aggregate mid-size expressed in fold improvement compared to cells in suspension; (**c**) Manually generated heterogeneous aggregates non-treated and (**d**) treated with invasive violacein producing bacteria. The lower panels exhibit propidium iodide staining of those aggregates. The scale bars represent 50 μm.

Background apoptosis in our conditions was measured as high as 10% by caspase activity staining and FACS analysis in HeLa S3 cells treated with invasive bacteria alone. We sought to increase the signal to noise ratio and circumvent that background apoptosis which should far out-compete the expected rate of positive hits. To alleviate this issue, we devised a strategy to screen the effect of metagenomic libraries against cell aggregates (Figure 7). We hypothesized that if an individual aggregate were to be dosed with drug producing bacteria, one would be able to more readily discern the presence of a true positive. It is expected that most of the cells of an aggregate will be affected by pro-apoptotic compounds, while background apoptosis should be randomly distributed across the aggregates (Figure 7a). In the case of a rate of 10% of background apoptosis, an average of 1 cell should be apoptotic in aggregates of ten cells, and the distribution of number of apoptotic cells within aggregates should follow a Poisson’s distribution. In theory, the larger the aggregates the greater the enhancement of signal over noise ratio (Figure 7b). 

Some transformed cell lines tend to grow as cell aggregates or readily form aggregates if placed in suspension, examples include the mouse embryonic carcinoma (EC) cell line P19 [58], human retinoblastoma cell line Y79 [59], and human medullary thyroid carcinoma cell line MTC-SK [60]. P19 EC cells were chosen because they have been used to generate embryoid bodies by culturing in non-tissue culture treated bacterial petri dishes [61] or by using the hanging drop method [62].

**Figure 8:**
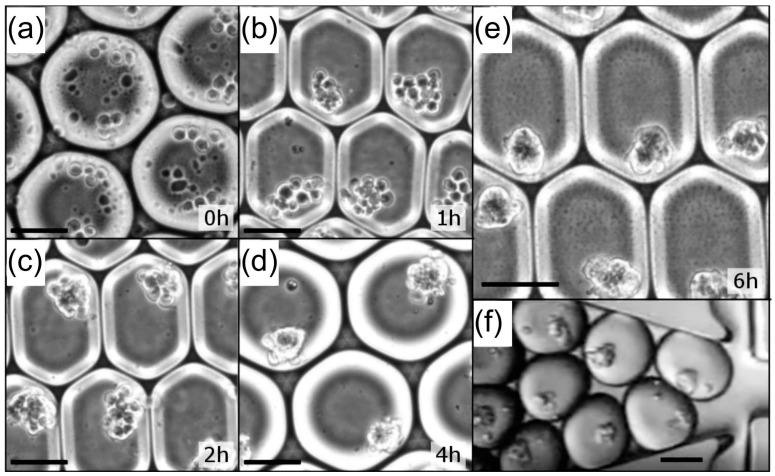
Formation of P19 EC (an average of 12 cells) aggregates into 110 μm (700 pL) droplets. Condensation of P19 EC cells over time in microfluidic droplets: (**a**) just after encapsulation; (**b**) 1 h; (**c**) 2 h; (**d**) 4 h and (**e**) 6 h incubation after encapsulation; (**f**) Those aggregates can then be re-injected to be combined with monoclonal droplets. Scale bars represent 50 μm.

It was clear when generating embryoid bodies via culturing P19 EC cells in bacterial petri dishes that it was possible to distinguish embryoid bodies treated with violacein producing DH10B versus controls even with a population heterogeneous in size (Figure 7c,d). We generated uniform aggregates by encapsulating an average of 12 cells into 110 μm (700 pL) droplets. This cell number was chosen to maximize the aggregate surface while minimizing the number of cells within its core. P19 EC cells started aggregating within a couple of hours and the aggregates were fully condensed within 6 h (Figure 8, Video S3). The aggregates exhibited homogenous sizes and minimum cell death (Figure S7). We could fully manipulate those aggregates and they were re-injected (Figure 8f, Video S4) into our merging chip to be combined with a bacterial library containing monoclonal droplets of invasin+pUC19-VioA-E+ DH10B and control invasin+ DH10B at a ratio of 1:1000. After infection of P19 EC aggregates, droplets were disrupted in bulk and aggregates were washed and incubated overnight. To both minimize loss of aggregates due to adhesion to plastic and aggregate-to-aggregate fusion, the aggregates were suspended in 0.1% ultra-pure agarose solubilized in cell culture media. Using Complex Object Parametric Analyzer and Sorter (COPAS) large particle flow cytometry [31], we sorted P19 EC aggregates positive for propidium iodide staining along with unselected aggregates. Lysing approximately 500 aggregates per condition only yielded ~20 colonies each, but 3 of the colonies recovered in the high-PI sorted group were violacein producing colonies. These results demonstrated a significant improvement in signal to noise ratio compared to the experiment with single HeLa S3 cells in suspension where only 9 out of 300 of the bacterial colonies recovered were positive for violacein. We could recover 500 negative aggregates without any false negatives: all the bacteria recovered from negative aggregates lacked pigmentation and thus were negative for violacein production. In addition to establishing a foundation for aggregate screening, this experiment demonstrated that issues with traditional in-plate screening such as loss of cell adhesion of apoptotic cells did not interfere with sorting and recovery of positive aggregates within the timeframe of the workflow.

## 4. Discussion

Of the 850 drugs on the market in 2008, at least 60% originated from nature or derived from natural compounds [63]. Culture of soil containing microbes has historically led to the well-known immunosuppressant rapamycin and to the discovery of a strain of *Streptomyces peucetius* that was modified to produce the widely used chemotherapeutic, doxorubicin [64,65]. More recently, exploiting the “unculturable” soil microbiome has provided the antibiotics teixobactin [66] and sansanmycin [67]; however, innovative approaches are needed to identify compounds with novel activities in mammalian cells at high throughput. This is the first report of a system for high throughput mammalian cell screening for metagenomic derived bio-active compounds. The absence of a method to directly connect biosynthetic clusters and their products to functional effects in mammalian cells has been an obstacle to true high throughput screening for metagenomic derived natural compounds. By utilizing invasive bacteria, we could link a biosynthetic gene cluster responsible for producing a compound (violacein) that led to the induction of apoptosis in target mammalian cells. The first aspect of our work involved rendering invasive a non-pathogenic *E. coli* DH10B and demonstrating that a bacterium could indeed be designed as a drug delivery vehicle for the poorly soluble violacein.

Microbial operons coding for enzymes involved in the complete synthesis of active bioproducts can vary widely in length. For example, the biosynthetic clusters for zwittermicin and epothilone span >60 kb [68,69]. However, metagenomic libraries comprised of smaller inserts still potentially encompass entire biosynthetic pathways. In our study, we worked solely with the violacein operon of *C. violaceum* which is a modest 8 kbp [52]. Other small to mid-range metagenomic DNA fragments (30 kb maximum) have also proven to be of value. Turbomycin A [15], for example, is synthesized from a biosynthetic cluster of less than a few kbp that was isolated from screening a metagenomic library of less than 25,000 clones in an *E. coli* host. Overall, a comprehensive strategy to realize the full potential of our platform would include libraries whose inserts would span the entire spectrum, i.e., a library with inserts averaging 20–25 kbp in length expressed in a high copy plasmid vector and a library with 100–150 kbp inserts cloned into a low copy BACmid.

Interestingly, several natural compounds such as turbomycin A and prodigiosin operon from *Janthinobacterium lividum* [70] were first identified based on their ability to affect the pigmentation of bacterial colonies. In variation of these studies, Arcamone et al. mutated *Streptomyces peucetius* producing daunorubicin with N-nitroso-N-methyl urethane to generate a novel isolate producing doxorubicin [64,65]. Once again, identification of the bacterial clone producing a drug variant was initially based on an obvious difference in pigment, and not function. It may very well be the case that there were novel modifications that resulted in doxorubicin-derived compounds with greater efficacy and lowered toxicity, but that those compounds went undetected because of a lack of alteration in pigment. These studies for drug mining and drug modification would suggest that potentially useful compounds that lack pigment are simply passed by due to a lack of high throughput functional assays. Therefore, both types of screens warrant the use of our unbiased functional assays. 

Our platform critically relies on the ability to recover intact invading bacteria for downstream analysis. Mammalian cells would phagocytose bacteria and degrade them in a series of specialized compartments through regulated processes and pathways. We observed for instance that we would recover a number of bacteria lower than the original input from HeLa S3 cells infected with invasin+ *E. coli*; however, the HeLa S3 cells infected with invasin+ violacein+ engineered *E. coli* had substantially higher numbers of *E. coli* even though much fewer HeLa cells were left adhering to the tissue culture plastic. We can only surmise that the process of endocytic degradation of the *E. coli* is impeded during apoptosis induced by violacein. This outcome may be related to the observation that bacteria can disrupt normal trafficking that usually results into their degradation in macrophages [71]. This was an unforeseen but very useful attribute to our screening approach. We also noted that the presence of DMSO in the recovery buffer was harmful to the recovered bacteria. Interestingly, as noted above, non-apoptotic producing bacteria tend to not survive the infection step thus reducing the number of false positive bacteria.

The second critical aspect of our platform consists of the screening technology based on droplet microfluidics. This technology enables the high-throughput manipulation and screening of large metagenomic libraries. It permits the unbiased amplification of metagenomic bacterial libraries, and the high-throughput combination of those libraries with target mammalian cells. Finally, we could demonstrate that using cell aggregates generated with droplet microfluidics permits to reduce the effect of background apoptosis and increase the rate of positive hits in large scale screens. It is the first time that microfluidic droplets have been used for clonal amplification of bacterial libraries and the controlled generation and manipulation of cell aggregates.

Our results with P19 embryonic carcinoma cells are highly encouraging and validate an approach where cell aggregates could be used to screen for anticancer compounds more efficiently. In particular, multiple primary cells and cell lines of pharmaceutical value, including mesenchymal stem cells [72], dopaminergic neurons [73], insulin producing pancreatic beta cells [74], as well as other cell types derived from normal tissues that would not perform well as single cells in solution, undergoing programmed cell death in suspension [75], could benefit from using aggregates. However, the aggregate approach is still limited to cell types that naturally create cell clusters. A way to circumvent this issue and make our technology more widely applicable to non-clustering cells would involve growing adherent cell lines on micro-carrier beads [76]. Interestingly, microcarriers have been utilized not only for the mere maintenance of viability of various cell lines but also for the active process of cell differentiation, suggesting their use for metagenomic drug screens of commercial value [77,78]. 

We have assumed a background apoptosis of 10% which is the highest rate we observed due to non-producing but invasive bacteria as measured by FACS experiments. Those data were obtained with cells in solution and the background rate could be expected to be higher when using droplet microfluidics because of the electrofusion module, interaction with the surfactant or addition of LB medium used to grow bacteria in droplets. The electrofusion module has already been used without any noticeable effect on cell viability [32]. Additionally, we observed only very limited apoptotic cells in the P19 EC cell aggregates generated as shown in Figure S7, which indicates a very good biocompatibility of the oil-surfactant system in the rather short timeframe of the experiments. Similarly, the effect of LB medium on encapsulated cells is limited by the short incubation time used to perform the infection which was allowed to occur between 30 min and 1 h, and by the fact that the LB medium was diluted more than 4-fold due to the merging of unequal droplets. Those factors, even unnoticeable separately, may contribute to the apoptosis background and would require further investigation to tease out their additive impact.

Drug leakage and shuffling between droplets is often overlooked in applications of droplet microfluidics. In our platform, the delivery of violacein through invasion was not tightly controlled and some of the compound was released within the medium when the bacteria were grown in bulk and was likely released within the monoclonal droplets when allowed to grow. We have observed that hydrophobic molecules can partition into the oil phase [32], certainly through micellar transport, with very little amount being transported into empty droplets and thus unlikely to trigger apoptosis in our case. Only compounds with activity at very small concentrations such as catalysts have been shown to elicit reactions in neighboring droplets [1,2]. In our dilution assays the violacein producing bacteria represented a very small fraction, which would be even more the case in a metagenomic screen, and would not be able to produce enough violacein in solution to affect hundreds of thousands of droplets. Finally, the absence of false negative confirms that the discussed factors cannot explain all the cell apoptosis observed using microfluidic droplets and that intracellular delivery of violacein does induce cell apoptosis.

A current limitation that would still need to be addressed in our workflow is the relatively low throughput of the sorting step when using large particles or aggregates. The COPAS technology used in this study reaches a maximum sorting rate of 100 events per second. A way to overcome this limitation would be to avoid cell sorting altogether and to rely on methods such as magnetic beads that bind to a cell surface epitope that is only present on positively affected cells. This has been previously done in the case of early stage apoptotic cells by repurposing annexin V binding magnetic beads originally designed to remove dead cells from culture [79]. For a wider range of applications, not just limited to apoptosis, the events could be sorted directly from droplets whose sorting performance compete with traditional FACS machine with throughput up to 30 k events per second [80,81,82].

In conclusion, we are reporting a promising platform to efficiently screen metagenomic libraries for pro-apoptotic natural compounds in mammalian cells by combining a novel bacterial delivery method and high-throughput droplet microfluidics. Specifically, we demonstrated that invasive bacteria could efficiently deliver a newly synthesized natural compound directly into mammalian cells. Finally, this platform was enabled by the droplet microfluidic technology which permits to amplify bacterial libraries with a minimal bias, and to generate homogeneous cell aggregates that allow for an increased mining efficiency of pro-apoptotic compounds.

## Supplementary Materials

The following are available online at www.mdpi.com/2072-666X/8/8/230/s1, Figure S1: Vector TRIP 3.0, Figure S2: Violacein as a model of a pro-apoptotic natural product, Figure S3: Effect of bacterial delivery of Violacein into Vero cells at MOI of 10, Figure S4: Caspase 8 staining of DH10B-VioA-E infected HeLa S3 cells, Figure S5: Effect of 10 μM purified violacein on HeLa S3 cells after a 10-h incubation, Figure S6: Sorting of HeLa S3 treated within monoclonal droplets, Figure S7: P19 EC cell aggregates generated by incubation in microfluidic droplets, Video S1: Generation of monoclonal droplets. For the purpose of the video, a concentrated solution of *E. coli* DH10B was encapsulated into 75 μm microfluidic droplets, Video S2: Merging of a monoclonal library with freshly encapsulated HeLa S3 cells using an electro-fusion module. The bacteria in this video carry a Red Fluorescent Protein reporter for visualization purpose, Video S3: Generation of P19 EC aggregates using microfluidic droplets, Video S4: Merging of a monoclonal library with encapsulated P19 EC aggregates using an electro-fusion module, File S1: CAD designs of microfluidic devices.
