# Supplementary material for: A Droplet Microfluidics Based Platform for Mining Metagenomic Libraries for Natural Compounds
Source: Micromachines (Basel). 2017 Jul 25;8(8):230. doi: 10.3390/mi8080230 (PMC6189830; doi:10.3390/mi8080230)
Supplement: Supplementary file 1 [file micromachines-08-00230-s001.zip › micromachines-205464-supplementary/micromachines-205464-supplementary.pdf]

A Droplet Microfluidics Based Platform for Mining Metagenomic Libraries for Natural Compounds

Elias Theodorou, Randall Scanga, Mariusz Twardowski, Michael P. Snyder and Eric Brouzes

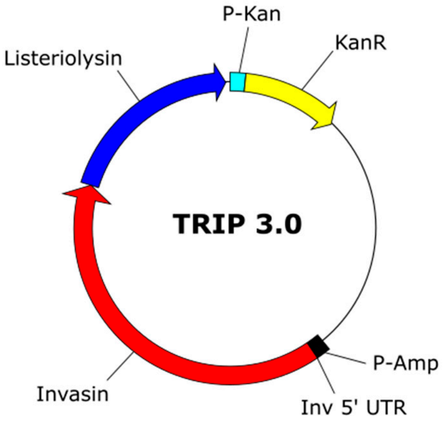

Figure S1. Vector TRIP 3.0. The P-Amp promoter drives dual expression of invasin and lysteriolysin. Invasin expression allows for internalization by mammalian cells expressing beta1-integrin and listeriolysin disrupts the integrity of phagocytic vesicles for efficient release of the contents from lysed bacteria into the cytosol.

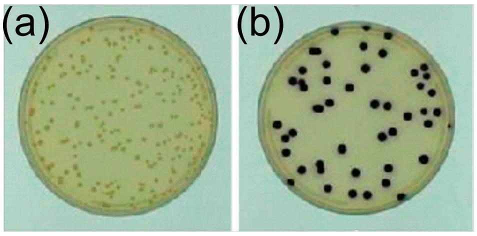

Figure S2. Violacein as a model of a pro-apoptotic natural product. (**a**) Invasive bateria form colorless colonies; (**b**) Violacein expression in invasive E.coli results in dark, pigmented colonies that allowed for trackable expression.

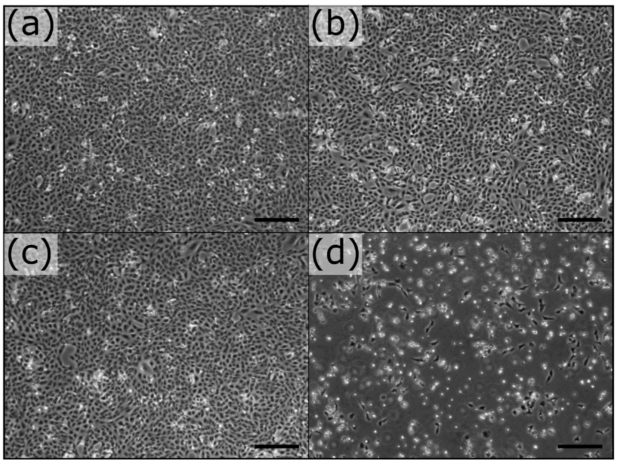

Figure S3. Effect of bacterial delivery of Violacein into Vero cells at MOI of 10. (**a**) Negative control; (**b**) Vero cells treated with inv+ only bacteria; (**c**) Vero cells treated with inv+ and empty pUc vector bacteria; (**d**) Vero cells treated with invasin bacteria expressing violacein exhibit a high level (about 94%) of cell loss. Scale bars represent 100 μm.

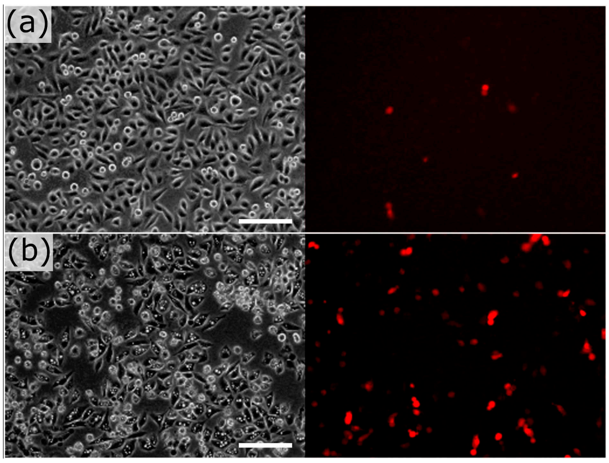

Figure S4. Caspase 8 staining of HeLa S3 cells infected with invasive only bacteria (**a**) and invasion violacein-expressing bacteria (**b**) at a MOI of 30 for 6 hours. The right panels indicate a much higher level of apoptosis in cells treated with the invasive violacein expressing bacteria (31% versus 3% of the cells). HeLa S3 cells treated with the invasive violacein expressing bacteria exhibited vacuoles in 64% of the cases; however, there was no clear correlation between the presence of vacuoles and apoptosis. Scale bars represent 100 μm.

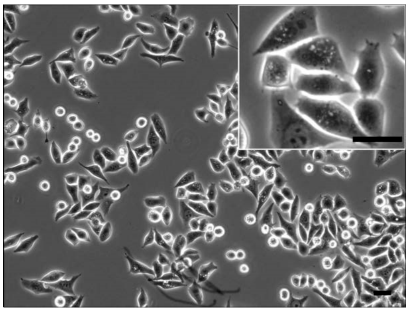

Figure S5. Effect of 10 μM purified violacein on HeLa S3 cells after a 10-hour incubation. The treated cells exhibit smaller vacuoles than cells treated with Violacein delivered by bacteria. The inset shows a zoomed in portion of the larger view image. Scale bar represents 20 μm.

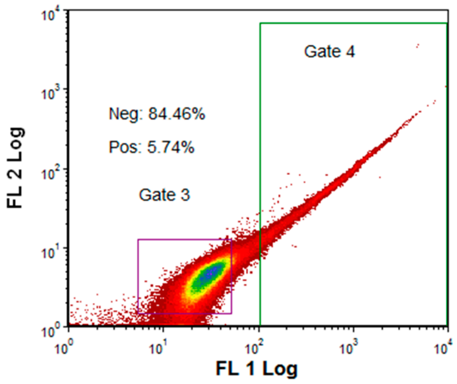

Figure S6. Sorting of HeLa S3 treated within monoclonal droplets.

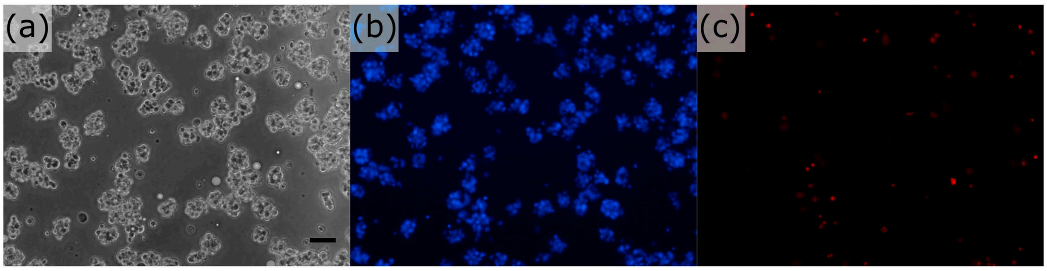

Figure S7. P19 EC cell aggregates generated by incubation in microfluidic droplets. (**a**) Phase contrast, (**b**) Dapi nuclear stain, and (**c**) Propidium Iodide staining for cell viability. Characterization of P19 EC aggregates confirm their homogeneity and viability.
